# Building the infection prevention workforce: development, implementation and evaluation of Texas Epidemic Public Health Institute infection prevention and control 300 series

**DOI:** 10.3389/fpubh.2026.1774483

**Published:** 2026-04-20

**Authors:** Kayla E. Ruch, Janelle Rios

**Affiliations:** 1 Texas Epidemic Public Health Institute Infection Prevention and Control Program Manager, School of Public Health, UTHealth Houston School of Public Health, Houston, TX, United States; 2 Environmental and Occupational Health Sciences, Texas Epidemic Public Health Institute, UTHealth Houston School of Public Health, Houston, TX, United States; 3 Texas Epidemic Public Health Institute, The University of Texas Health Science Center at Houston, Houston, TX, United States

**Keywords:** continuing education (CE), healthcare epidemiology, infection preventionists, IPC, public health workforce

## Abstract

**Background:**

The Texas Epidemic Public Health Institute (TEPHI) was established to strengthen infectious disease preparedness across Texas. The Infection Prevention and Control (IPC) 300 Series represents the third year of a multi-tiered educational initiative designed to provide free, accessible, evidence-based IPC training and continuing education credits for the healthcare workforce.

**Methods:**

Registration and attendance data were collected through Zoom™ and Microsoft Teams™. Knowledge assessments, post-module surveys, and mid- and end-of-series evaluations were administered through QuestionPro™. Module content was informed by guidance from APIC, CDC, CMS, and OSHA. Descriptive statistics and the Kirkpatrick Model of Training Evaluation were used to assess participant reaction, knowledge acquisition, and intended behavior change. Quantitative analyses were conducted in Stata/BE 18.0.

**Results:**

The 300 Series registered 5,230 individuals, including 1,685 live attendees and 3,698 asynchronous YouTube views. Each module qualified for 1 h of continuing education credit across multiple certifications (a-IPC, CIC®, CPH, CME, CNE). Among 511 participants completing knowledge assessments, the mean score was 92% (95% CI: 90.8–93.2), exceeding the 80% competency threshold. Participants reported high satisfaction across clarity, usefulness, and relevance (mean 4.7/5.0). A total of 91.4% of respondents planned to apply the knowledge gained, and 97.3% intended to participate in future modules.

**Conclusion:**

The TEPHI IPC Seminar Series supports IPC workforce development by delivering accessible, no-cost, competency-based training. Outcomes from 2023 to 2025 demonstrate strong program reach, high participant satisfaction, and positive learning outcomes across the first three levels of the Kirkpatrick Model. These findings suggest that scalable, competency-based virtual training models may represent a promising strategy for strengthening infection prevention workforce capacity and public health preparedness.

## Introduction

Infection prevention and control (IPC) is a foundational component of patient safety and healthcare quality, grounded in evidence-based strategies that reduce the transmission of infectious agents, protect healthcare workers, and prevent avoidable harm (1). Effective IPC requires coordinated efforts across healthcare systems, public health agencies, and regulatory bodies to ensure that policies, surveillance systems, and prevention strategies are consistently implemented (1). As a multidisciplinary field, IPC encompasses key domains such as healthcare-associated infection (HAI) prevention, outbreak detection and response, environmental hygiene, sterilization and disinfection, occupational health, antimicrobial stewardship, and the promotion of a strong safety culture (1).

HAIs remain a substantial global threat, contributing to preventable morbidity, mortality, and escalating healthcare costs—particularly in settings with limited water, sanitation, and hygiene (WASH) infrastructure (2). The World Health Organization’s Second Global Report on IPC highlights that up to 70% of HAIs may be preventable with strong IPC and WASH programs and that investments in IPC yield significant returns by improving patient outcomes and reducing antimicrobial resistance (2). Epidemic-prone pathogens such as Ebola, Marburg virus, Mpox, and COVID-19 further underscore the vulnerability of healthcare systems and the critical need for a well-prepared IPC workforce capable of responding rapidly to emerging threats (3). On average, 7% of hospitalized patients in high-income countries and 15% in low- and middle-income countries acquire at least one HAI, and without strengthened IPC systems, HAIs could contribute to as many as 3.5 million deaths annually by 2050 (3).

Despite global progress, IPC training and workforce development remain inconsistent. Although 91% of countries report national HAI prevention guidelines, only 38% have established formal IPC curricula, resulting in variation in training quality and competency among infection preventionists (3). IPC professionals—who often enter the field from nursing, public health, laboratory science, or allied health backgrounds—are responsible for surveillance, outbreak investigations, policy development, and staff training across diverse clinical settings (4). As the healthcare system grows more complex and the demand for IPC expertise increases, structured and scalable educational pathways are urgently needed.

Texas exemplifies these challenges. Home to nearly 32 million residents and projected to exceed 36–44 million by 2060 (5), the state faces significant pressures on healthcare capacity and workforce preparedness. To address some of these gaps, the Texas Legislature established the Texas Epidemic Public Health Institute (TEPHI) through Senate Bill 1780, housed at UTHealth Houston School of Public Health. TEPHI was charged with strengthening statewide infectious disease preparedness through workforce development, communication, and evidence-based training (6). In response, TEPHI developed the Infection Prevention and Control (IPC) Webinar Series, a free, virtual, statewide educational program designed to strengthen IPC capacity, particularly in rural, underserved, and resource-limited settings (7, 8).

The series is structured across three tiers—Foundational IPC Principles (100 Series, 2023), Applied Epidemiology and Program Management (200 Series, 2024), and Clinical and Systems-Based IPC Applications (300 Series, 2025). Together, these modules provide a comprehensive, competency-aligned pathway to support early-career professionals, cross-trained staff, and experienced IPC practitioners seeking continuing education. This paper aims to (1) evaluate the third-year performance of the TEPHI IPC 300 Series, (2) analyze its development, reach, and growth across the three program years, and (3) provide recommendations for advancing future IPC education and research. The present evaluation examines program implementation, reach, and educational outcomes, including participation metrics, learner satisfaction, knowledge acquisition, and participants’ reported intention to apply IPC concepts. Outcomes such as long-term behavioral change, institutional practice adoption, or infection prevention outcomes were outside the scope of this analysis. The evaluation framework was guided by the Kirkpatrick Model of Training Evaluation, which assesses participant reaction, learning outcomes, and reported behavior change following workforce training interventions.

## Materials and methods

This analysis followed the same methodological framework described in the prior 100- and 200-Series publications (7, 8). The TEPHI IPC Series was developed using the eight Certified in Infection Control (CIC®) core competency domains defined by the Certification Board of Infection Control and Epidemiology (CBIC) (9). These domains represent the industry standard for assessing infection preventionists’ proficiency in: (1) identification of infectious disease processes, (2) surveillance and epidemiologic investigations, (3) prevention and control of infectious agent transmission, (4) employee and occupational health, (5) management and communication, (6) education and research, (7) environment of care, and (8) cleaning, sterilization, disinfection, and asepsis (9). Content was developed with guidance from best practices and recommendations published by the Association for Professionals in Infection Control and Epidemiology (APIC), Centers for Disease Control and Prevention (CDC), Centers for Medicare and Medicaid Services (CMS), and Occupational Safety and Health Administration (OSHA).

This study employed a non-experimental, one-group post-test program evaluation design to assess the implementation and performance of the TEPHI IPC 300 Series. Because the evaluation relied on post-session assessments without baseline measurement or a comparison group, causal inferences regarding program impact cannot be made. The evaluation examined program reach, participant reaction, learning outcomes, and intended behavior change using the Kirkpatrick Model of Training Evaluation (10). The primary evaluation questions were: (1) What was the reach and participation of the program? (2) How did participants evaluate the relevance and quality of the training? (3) To what extent did participants demonstrate knowledge acquisition through module assessments? and (4) Did participants report intent to apply IPC practices learned during the training?

Evaluation indicators were selected based on the Kirkpatrick Model of Training Evaluation, a widely used framework for assessing workforce training programs. Participation metrics assessed program reach, knowledge assessment scores evaluated learning outcomes (Level 2), and post-module survey items measured participant reaction and intended behavior change (Levels 1 and 3). Together, these indicators capture key dimensions of program implementation and early educational outcomes.

The 300 Series was implemented from February to November 2025 and comprised ten monthly modules designed to deliver evidence-based IPC education across diverse healthcare and public health settings in Texas. Table 1 provides an overview of module content, objectives, and references. The target audience included individuals identifying as infection prevention professionals or those responsible for their facility’s IPC program. Although the primary audience was Texas participants, the series was open nationally and internationally. Each session was delivered synchronously via Zoom™ (11) and Microsoft Teams™ (12), with recordings made publicly available on the TEPHI YouTube channel to facilitate asynchronous learning, statewide access, and refresher training.

**Table 1:** Infection control series module overview.

Module number and title	Overview	Learning objectives	Additional material
Module 301: central line- associated bloodstream infections (CLABSIs)	We will introduce CLABSIs, discuss reduction efforts and patient engagement with CLABSI prevention.	(1) Describe central line-associated bloodstream infections (CLABSIs) (2) Discuss CLABSI prevention measures (3) Describe and discuss patient engagement for Prevention	APIC textbook, CDC and NHSN (22)
Module 302: *Clostridioides difficile* infections (CDIs)	This introduction will briefly outline CDIs, discuss efforts for their reduction and prevention, and explore strategies to engage patients in prevention initiatives.	(1) Describe *Clostridioides difficile* infections (CDIs) (2) Discuss CDIs prevention measures (3) Describe and discuss patient engagement for prevention	APIC textbook, CDC and NHSN (23)
Module 303: infection prevention and control training	This module provides a brief overview of infection prevention and control training for infection preventionists, nurses, clinicians, and ancillary healthcare workers	(1) Describe and discuss infection prevention and control (IPC) training for IPs (2) Describe and discuss IPC training for nurses and clinicians (3) Describe and discuss IPC training and education for ancillary staff	APIC, CBIC, CDC, NIOSH, OSHA and WHO (24)
Module 304: healthcare environment	An overview of the environment of care (EOC), including key components and regulatory expectations, with a focus on conducting EOC risk assessments	(1) Describe and discuss the environment of care (EOC) (2) Discuss EOC risk assessments (3) Discuss the joint commission (TJC) risk assessments	APIC textbook, CDC and TJC (25)
Module 305: survey readiness	This lecture module will provide a brief overview of survey readiness, focusing on IPC survey readiness, the annual plan, and risk assessment.	(1) Describe and discuss survey overview (2) Describe and discuss IPC survey plan (3) Describe and discuss IC annual plan/risk assessment	APIC textbook, CDC, and TJC (26)
Module 306: high-level disinfection	A brief introduction to healthcare high-level disinfection practices and monitoring	(1) Define and describe the foundations high-level disinfection (HLD) (2) Describe HLD process for specific equipment (3) Describe and discuss HLD monitoring processes	APIC Textbook, ARON, CDC, and FDA (27)
Module 307: sterilization	This lecture module will provide a brief introduction to healthcare sterilization, practices, and monitoring	(1) Define and describe foundational components of sterilization (2) Understand the sterilization process (3) Apply monitoring sterilization practices	ANSI/AAMI, APIC Textbook, CDC, TJC, and WHO (28)
Module 308: emerging infectious pathogens	This lecture module will provide a brief overview of the 2024 Marburg outbreak in Rwanda, along with updates on Dengue and H5N1 activity in the United States.	(1) Discuss the 2024 Rwanda Marburg outbreak (2) Discuss Dengue status in the United States (3) Discuss H5N1 status in the United States	APIC Textbook, CDC, DSHS, and WHO (29)
Module 309: outbreak investigation	This lecture module will provide a brief overview of how to conduct an outbreak investigation in acute-care settings, the role of state health departments, and the importance of collaboration during patient transfers to long-term care facilities.	(1) Explain the outbreak investigation process in an acute-care setting, illustrated through a *Candida auris* case study. (2) Identify the responsibilities of DSHS during an outbreak investigation in a healthcare facility. (3) Discuss the outbreak investigation process in long-term care facilities and explain why interfacility communication is important.	APIC Textbook, CDC, DSHS, and EPA (30)
Module 310: IPC in special populations	This lecture module will provide a brief introduction to infection prevention and control practices (IPC) in special populations within healthcare settings.	(1) Define and discuss IPC in behavioral health populations (2) Define and discuss IPC in pediatric populations (3) Define and discuss IPC in outpatient settings	APIC Textbook, CDC, and OSHA (16)

Registration and attendance metrics were collected through Zoom™ and Microsoft Teams™. YouTube analytics tracked asynchronous viewership. Continuing education (CE) credits eligibility included Associate in Infection Prevention and Control (a-IPC), Certification in Infection Control (CIC®), Certified in Public Health (CPH), Continuing Medical Education (CME), Continuing Nursing Education (CNE), Certified Health Education Specialist (CHES), and Registered Sanitarian (RS) credits.

Knowledge assessments consisted of 10 multiple-choice questions embedded within each module. Questions assessed comprehension of key IPC concepts presented during the session. A score of ≥80% was required to obtain continuing education credit, consistent with standard CE competency thresholds. Assessments were designed to evaluate post-session knowledge acquisition rather than baseline knowledge. CE completion was tracked through the program registration and evaluation platform. Knowledge assessments measured comprehension corresponding to Kirkpatrick Level 2 (Learning).

Post-module evaluations included Likert-type items assessing clarity, usefulness, scope, and intent to implement content (Kirkpatrick Levels 1 and 3), along with open-ended questions on educational needs and feedback administered via QuestionPro™ (13). Complete case analysis was used to examine demographic data and post-survey evaluations. Mid-series (mid-July) and end-of-series (mid-October) evaluations were distributed to all registered participants to assess program quality, identify IPC challenges, and guide ongoing module development. Additionally, beginning in August 2025, a monthly virtual networking forum was established to promote professional engagement, exchange best practices, and foster peer-to-peer support.

Descriptive statistics summarized registration, attendance, viewership, knowledge scores, CE participation, and survey responses. Quantitative analyses were performed using Stata/BE version 18.0 (14). To evaluate trends across program years (2023–2025), measures of central tendency and pairwise t-tests were used, assuming equal variance and statistical significance at *p* < 0.05. Comparisons across program years used independent-sample t-tests because participation differed across cohorts and repeated measures at the individual level could not be confirmed. Although Likert-scale responses represent ordinal data, parametric tests were used due to the large sample size and the common application of these approaches in program evaluation research.

Open-ended responses were analyzed using an inductive thematic analysis approach informed by Braun and Clarke’s methodology (15). Responses were exported into Microsoft Excel and reviewed in multiple stages. First, all responses were read in full to achieve familiarization with the data. Second, initial codes were generated through line-by-line review, capturing key concepts and recurring ideas. Codes were then grouped into higher-order categories and iteratively refined into overarching themes representing participant-reported challenges and educational needs. A coding framework was developed and applied consistently across responses. Frequencies of themes were summarized descriptively to identify the most reported barriers (e.g., time constraints, resource limitations, organizational resistance). Representative responses were used to support thematic findings.

This multi-year project was funded by the 87th Texas Legislature (2021 Regular Session). The evaluation did not involve identifiable information and was conducted for the purpose of training program improvement. As such, it did not meet criteria for human subjects research and did not require Institutional Review Board (IRB) review.

## Results

A total of 5,230 individuals registered for the TEPHI IPC 300 Series, with 1,685 participants attending sessions synchronously and more than 3,698 engaging asynchronously through YouTube (Table 2). Although 61.4% of registrants (*n* = 3,208) reported a Texas residence, participation extended nationally across 49 U.S. states and internationally to more than 20 countries. Participants claimed 346 continuing education (CE) credits by March, 13, 2026. Participation varied across modules, with Module 308: Emerging Infectious Pathogens generating the highest live attendance and knowledge assessment submissions, while Module 301: CLABSI had the lowest attendance. Module 303: Infection Prevention and Control Training had the highest number of YouTube views. Attendance also increased during the final third of the series, suggesting growing momentum and sustained interest over time.

**Table 2:** Module registration, attendance, and YouTube views.

Module	Date presented	Registrants	Attendees	YouTube views (3/13/26)	Total view (attendee+ YouTube)	Continued education eligibility
Module 301	06-Feb-2025	237	73	687	760	a-IPC, CIC, CPH
Module 302	06-Mar-2025	264	80	254	334	a-IPC, CIC, CPH
Module 303	03-Apr-2025	436	109	1,092	1,201	a-IPC, CIC, CPH
Module 304	02-May-2025	584	122	327	449	a-IPC, CIC, CPH CME, CNE, CHES, RS
Module 305	05-Jun-2025	567	116	174	290	a-IPC, CIC, CPH CME, CNE, CHES, RS
Module 306	03-Jul-2025	690	118	251	369	a-IPC, CIC, CPH CME, CNE, CHES, RS
Module 307	07-Aug-2025	649	105	339	444	a-IPC, CIC, CPH CME, CNE, CHES, RS
Module 308	04-Sept-2025	712	409	202	611	a-IPC, CIC, CPH CME, CNE, CHES, RS
Module 309	02-Oct-2025	601	309	236	545	a-IPC, CIC, CPH CME, CNE, CHES, RS
Module 310	06-Nov-2025	490	244	136	380	a-IPC, CIC, CPH CME, CNE, CHES, RS
Total		5,230	1,685	3,698	5,383	

Among the 5,212 participants who provided demographic information, the majority were aged 30–39 years (26.5%) or 40–49 years (26.3%), with smaller proportions aged 50–59 years (17.2%), 20–29 years (15.6%), and ≥60 years (8.8%) (Table 3). Most respondents identified as female (79.3%). Racial and ethnic representation included White (52.2%), Asian (14.6%), Black or African American (14.2%), and individuals reporting two or more races (5.3%), with 23.0% identifying as Hispanic or Latino. Professional experience varied widely; 41.7% of participants had been in their current role for 0–2 years, while 34.0% reported 3–5 years of experience. Infection prevention experience was similarly variable, with 30.5% reporting 0–2 years and 14.6% reporting more than 10 years. Notably, nearly one quarter (23.8%) were not currently working in designated IPC roles, indicating participation from cross-trained or transitioning professionals.

**Table 3:** Infection control series attendee demographic and occupational characteristics.

Characteristics	Series (*n* = 5,212)
Age	
<20	9 (0.2%)
20–29	813 (15.6%)
30–39	1,379 (26.5%)
40–49	1,371 (26.3%)
50–59	897 (17.2%)
60+	459 (8.8%)
Prefer not to answer	284 (5.4%)
Gender	
Female	4,132 (79.3%)
Male	846 (16.2%)
Prefer not to answer	234 (4.5%)
Race	
American Indian or Alaskan Native	57 (1.1%)
Asian	759 (14.6%)
Black or African American	741 (14.2%)
Middle Eastern or North African	119 (2.3%)
Native Hawaiian or Pacific Islander	15 (0.3%)
Two or more races	275 (5.3%)
White	2,721 (52.2%)
Prefer not to answer	525 (10.1%)
Ethnicity	
Hispanic or Latino	1,197 (23.0%)
Not Hispanic or Latino	3,367 (64.6%)
Prefer Not to Answer	648 (12.4%)
Years in current position	
0–2 yrs.	2,174 (41.7%)
3–5 yrs.	1,773 (34.0%)
6–10 yrs.	631 (12.1%)
10 + yrs.	634 (12.2%)
Years in infection prevention	
0–2 yrs.	1,587 (30.5%)
3–5 yrs.	1,034 (19.8%)
6–10 yrs.	590 (11.3%)
10 + yrs.	760 (14.6%)
Not in IPC	1,231 (23.8%)

**Figure 1:**
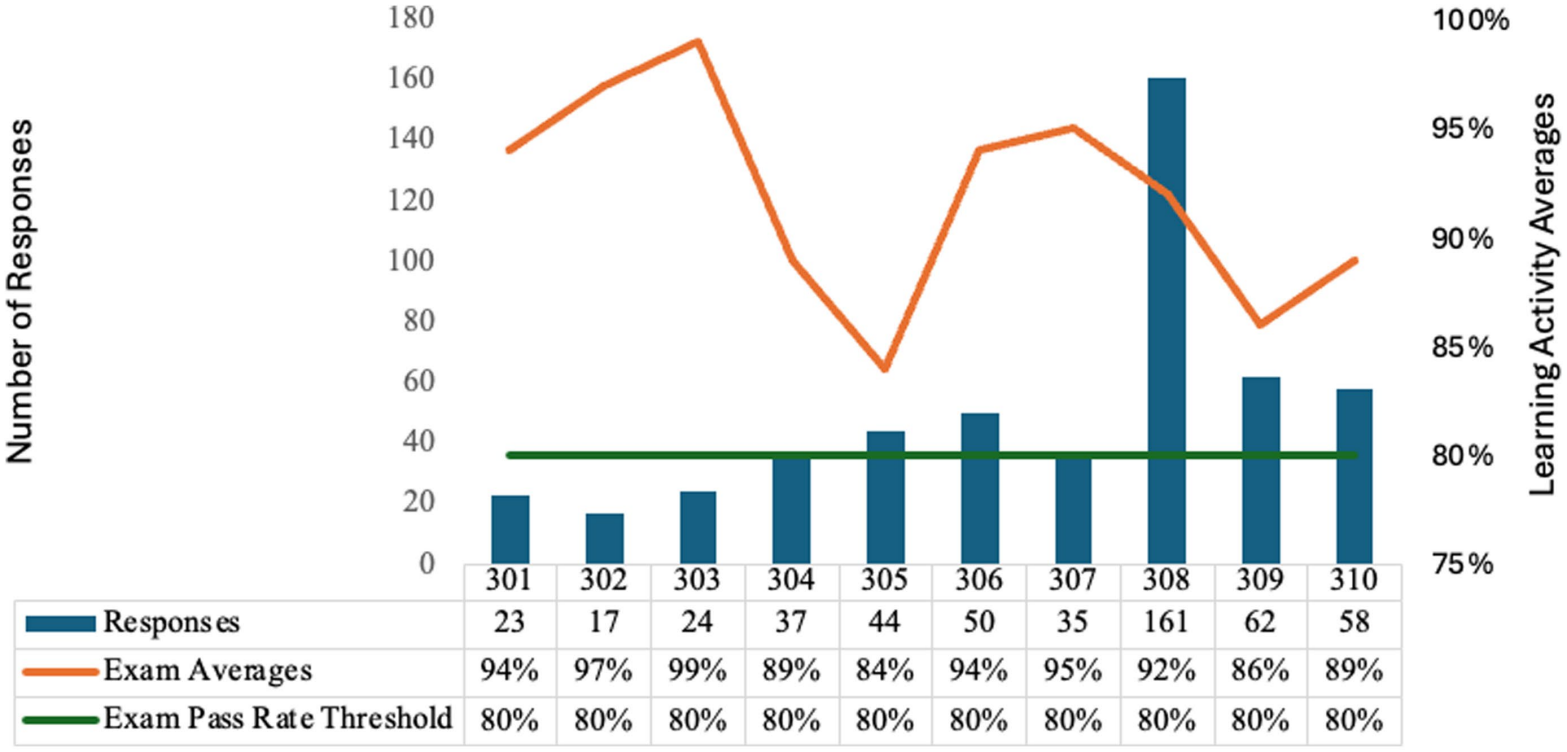
Module learning activity series overview.

Knowledge assessment data were available for 511 participants (9.8% of registrants) (Figure 1). Submission volume ranged from 17 assessments for Module 302: *Clostridioides difficile* Infection to 161 submissions for Module 308: Emerging Infectious Pathogens, mirroring module-specific attendance patterns. Mean scores ranged from 84 to 99% across modules, with an overall average score of 92%, exceeding the ≥80% CE credit threshold for all modules.

Post-module evaluations demonstrated consistently high satisfaction (Table 4). Among 478 respondents, 79.9–82.0% “strongly agreed” that module content was beneficial, clearly presented, easy to understand, and appropriately scoped, with an additional 14–17% selecting “agree.” “Neutral” responses were uncommon (1.9–2.9%), and “disagreement” was rare (<0.5%). Of the four participants who selected “strongly disagree” for any item, all nonetheless reported that the knowledge checks improved their understanding and that they intended to apply the material and attend future IPC offerings. Overall, 69.3% of respondents indicated that the module content had not been previously presented to them, while 88.3% reported improved understanding and 97.5% stated that the knowledge checks enhanced comprehension. Furthermore, 91.4% planned to implement the knowledge gained, and 97.3% expressed intent to attend future IPC seminars.

**Table 4:** Modular post-survey evaluations.

Modular post-survey evaluations	*n* (%) (*n* = 478)
The material presented was beneficial?	
Strongly Agree	382 (79.9%)
Agree	81 (17.0%)
Neutral	11 (2.3%)
Disagree	0 (0.0%)
Strongly Disagree	4 (0.8%)
The amount of content covered was appropriate?	
Strongly Agree	387 (81.0%)
Agree	78 (16.3%)
Neutral	9 (1.9%)
Disagree	0 (0.0%)
Strongly Disagree	4 (0.8%)
Was the material being easy to understand?	
Strongly Agree	391 (81.8%)
Agree	71 (14.9%)
Neutral	10 (2.1%)
Disagree	2 (0.4%)
Strongly Disagree	4 (0.8%)
Was the material clear and concise?	
Strongly Disagree	392 (82.0%)
Disagree	68 (14.2%)
Neutral	14 (2.9%)
Agree	0 (0.0%)
Strongly Agree	4 (0.8%)
Has this material been presented to you before?	
Yes	142 (29.8%)
No	330 (69.3%)
Prefer Not to Answer	4 (0.8%)
Did the module help improve your understanding of the topic(s) covered?	
Yes	422 (88.3%)
No	56 (11.7%)
Did the knowledge check activity help improve your understanding of the material?	
Yes	466 (97.5%)
No	12 (2.5%)
Will you implement the knowledge gained from this seminar at your organization?	
Yes	437 (91.4%)
No	1 (0.2%)
Prefer Not to Answer	40 (8.4%)
Do you plan to attend future infection prevention seminar series modules?	
Yes	465 (97.3%)
No	6 (1.3%)
Prefer Not to Answer	7 (1.5%)

When evaluating the 300 Series as a whole, series-wide evaluation results were consistent with module-level feedback. Of more than 5,000 individuals who received the end-of-series survey, 113 initiated and 101 completed it (2.0% completion rate). Among respondents, 80–82% strongly agreed that the 300 Series was beneficial, appropriate, and clearly presented, and 85.7% reported substantial improvement in their IPC understanding. Approximately 97.3% stated that the knowledge checks enhanced comprehension, and most participants reported applying or planning to apply concepts learned (44 and 52%, respectively). Thematic analysis of open-ended responses identified three primary barriers to implementation: time constraints, resource limitations, and organizational resistance. Despite these challenges, 95% intended to participate in future seminars, and 81% expressed interest in monthly networking sessions.

To support community building and peer learning, TEPHI hosted monthly networking sessions from August to December 2025. Across 5 months, 323 individuals participated, averaging 65 attendees per session. These sessions fostered professional dialogue, real-time problem solving, and continued learning. Networking sessions addressed topics such as infection prevention program implementation challenges, outbreak response coordination, surveillance practices, and peer-to-peer problem solving. These forums provided opportunities for participants to share practical experiences and discuss strategies for overcoming organizational barriers to IPC implementation.

Across the three-year series (2023–2025), participation and engagement increased steadily. The combined program registered 9,068 participants, with 3,357 live attendees and 12,761 asynchronous learners, totaling 16,118 learning encounters (Table 5). Registration approximately doubled between the 100- and 300-Series, synchronous attendance increased by 54.9%, and YouTube viewership grew consistently. Learner satisfaction remained high across all years, with mean evaluation scores exceeding 4.7/5.0 for all domains (Table 6). Pairwise t-tests revealed modest but statistically significant differences between Years 2 and 3 in perceived benefit, content appropriateness, and ease of understanding (*p* < 0.05), likely reflecting increased topic complexity in the 300 Series rather than diminished content quality. No significant differences were observed in intention to attend future seminars.

**Table 5:** TEPHI IPC series cumulative participation metrics (2023–2025).

Series	Year	Registrants	Attendees	YouTube views	Continuing education hours	Reach
100	2023	1,203	584	2,809	109	3,393
200	2024	2,635	1,088	6,254	220	7,342
300	2025	5,230	1,685	3,698	346	5,383
Total		9,068	3,357	12,761	675	16,118

Reach = Attendees and YouTube views. YouTube metrics represent total video view counts reported by the YouTube analytics platform and do not represent unique viewers.

**Table 6:** Comparison analysis between years 1, 2, and 3.

Evaluation item	Year 1 mean	Year 2 Mean	Year 3 mean	Comparison	*t*-statistic	*p* value
The material presented was beneficial	4.83	4.82	4.73	Y1 vs. Y2	0.22	0.83
				Y1 vs. Y3	2.30	**0.022**
				Y2 vs. Y3	2.99	**0.0029**
The amount of content covered was appropriate	4.76	4.84	4.75	Y1 vs. Y2	−1.73	0.085
				Y1 vs. Y3	0.23	0.82
				Y2 vs. Y3	2.99	**0.0029**
The material was easy to understand	4.80	4.84	4.75	Y1 vs. Y2	−0.86	0.39
				Y1 vs. Y3	1.15	0.25
				Y2 vs. Y3	2.99	**0.0029**
Will you implement the knowledge gained?	4.81	4.82	4.76	Y1 vs. Y2	−0.22	0.83
				Y1 vs. Y3	1.15	0.25
				Y2 vs. Y3	1.99	**0.047**
Do you plan to attend future seminars?	0.989	0.989	0.974	Y1 vs. Y2	0.00	1.00
				Y1 vs. Y3	0.34	0.73
				Y2 vs. Y3	0.50	0.62

Equal variance assumed, SD = 0.40; n₁ = 102, n₂ = 276, n₃ = 487. Degrees of freedom = n1 + n2–2 for each pairwise comparison. *p* < 0.05 indicates statistical significance (bold).

## Discussion

The findings from this three-year evaluation suggest that the TEPHI IPC Seminar Series represents a scalable model for strengthening the IPC workforce across diverse healthcare settings. The 300 Series alone reached more than 5,200 individuals in 2025 and contributed to more than 16,100 cumulative learning encounters across the three-year IPC series. These participation patterns reflect sustained and growing demand for accessible, evidence-based IPC education. These participation patterns mirror the current IPC landscape, in which professionals frequently assume IPC responsibilities without structured training—particularly in rural or resource-limited settings where IPC roles may be cross-trained or part-time (4, 5, 7). Although the program was designed to strengthen the Texas infection prevention workforce, participation extended nationally and internationally, with attendees representing 49 U. S. states and more than 20 countries. The program’s virtual delivery model and competency-aligned curriculum suggest that similar educational approaches may be adaptable to other jurisdictions seeking scalable IPC workforce development strategies.

Program outcomes aligned closely with the Kirkpatrick Model of Training Evaluation (10). At Level 1 (Reaction), participants consistently rated the modules as beneficial, clearly presented, and appropriately scoped, with high satisfaction maintained across all three program years. These findings suggest that the series successfully addressed key educational needs while leveraging a virtual format that reduces common barriers to professional development, including geography, cost, and limited institutional support—factors frequently cited in IPC workforce studies (4, 7).

At Level 2 (Learning), knowledge acquisition was reflected in a high average assessment score of 92%. The breadth of content—spanning clinical, epidemiologic, and systems-based IPC principles—enabled the series to engage practitioners across experience levels, as reflected in the wide range of roles and professional backgrounds observed in registrants. This is particularly relevant given persistent national training gaps; many infection preventionists report minimal formal IPC training and rely heavily on self-directed learning or informal mentoring (7, 8, 16). Recent U.S. evidence reinforces these concerns. Holmes et al. demonstrated that structured, competency-based education significantly improves certification preparedness and reduces variability in IPC knowledge (17), while Ruch et al. reported substantial variability in CIC® examination rates across institutions, emphasizing the need for accessible, standardized training pipelines (18). The strong learning outcomes observed in the TEPHI IPC Series provide additional support for structured, competency-aligned educational approaches.

At Level 3 (Behavior), participants reported strong intent to apply concepts learned (91.4%), and nearly half (44%) indicated that they had already implemented practices presented during the series. Although these outcomes were self-reported, they suggest potential early-stage behavior change, particularly given that most respondents had not previously encountered the material. Monthly networking forums may have further supported knowledge translation by facilitating real-time problem-solving, peer support, and discussions about implementation barriers. Participants cited organizational resistance, time constraints, and limited resources as common challenges—concerns consistently documented in IPC workforce assessments (17–21). This is consistent with the findings from the mid and end of series survey trend analysis. Despite these barriers, high ongoing engagement suggests that structured IPC education may strengthen both professional confidence and leadership identity among infection preventionists.

These findings also align with international research documenting substantial variability and fragmentation in IPC training. Studies in Australia and New Zealand identified wide disparities in IPC curricula, inconsistent competency expectations, and uneven support for national standards (20). Similarly, a WHO Eastern Mediterranean Region analysis highlighted large gaps in formal IPC training capacity and widespread reliance on informal, on-the-job learning (21). Texas-specific evidence mirrors these issues: a statewide survey found that many infection preventionists lacked structured onboarding, had uneven exposure to core competencies, and felt underprepared for CIC® certification (18). Taken together, these studies highlight a critical need for accessible, standardized, competency-based IPC education—needs that the TEPHI IPC Series was designed to address.

By aligning its curriculum with CIC® domains, offering interdisciplinary continuing education credits (CME, CNE, CHES, RS), and delivering content in a tiered structure (100–300 Series), the program provides a clear developmental pathway that benefits early-career professionals, cross-trained staff, and experienced practitioners seeking advanced training. Sustained high engagement across program years suggests that free, virtual, competency-based education can meaningfully supplement traditional IPC training models, including institutional onboarding and professional society offerings. As healthcare systems continue to face emerging pathogens, workforce shortages, and increasing complexity, scalable training strategies such as the TEPHI IPC Series may play an important role in strengthening infection prevention workforce capacity and public health preparedness.

### Strengths and limitations

Major strengths of this evaluation include its multi-year scope, large and diverse participant base, and use of a competency-aligned educational framework. The integration of embedded knowledge assessments and broad participation across urban, rural, and resource-limited settings enhances the program’s relevance to workforce development and public health preparedness.

Several limitations warrant consideration. Participation in the series and completion of evaluations were voluntary, introducing the potential for selection bias, as individuals with greater interest or prior engagement in infection prevention topics may have been more likely to participate and respond. The end-of-series evaluation had a low response rate (~2%), which may limit the representativeness of satisfaction and implementation findings. Behavioral outcomes were self-reported and may not reflect actual practice change within healthcare settings. Additionally, this evaluation did not assess facility-level or long-term outcomes such as healthcare-associated infection (HAI) reduction, limiting the ability to evaluate Kirkpatrick Level 4 outcomes. Because the evaluation relied on post-session assessments without baseline measurement or a comparison group, causal inferences regarding program impact cannot be made.

### Future directions

Learner feedback and networking forums identified several priority areas for future programming, including National Healthcare Safety Network (NHSN) surveillance, device reprocessing, multidrug-resistant organism (MDRO) prevention, behavioral health and pediatric infection prevention, ambulatory and outpatient IPC practices, vaccine hesitancy, occupational health, and IPC career development. Expanding module offerings or developing certificate-based tracks in these areas may further strengthen program reach and workforce preparedness.

Future research should evaluate longer-term outcomes associated with IPC workforce training, including sustained behavior change, organizational adoption of IPC practices, and potential effects on infection prevention outcomes. Although demographic characteristics were collected to describe the participant population, subgroup analyses were not conducted in this evaluation. Future studies should explore whether learning outcomes or implementation intentions vary by professional role, experience level, or IPC background. Longitudinal or facility-level analyses may also help determine whether structured IPC education is associated with improvements in institutional infection prevention practices or healthcare-associated infection outcomes.

## Conclusion

The TEPHI IPC Seminar Series supported IPC workforce development through an accessible, scalable, virtual, competency-based training model. Across 3 years, the series engaged more than 16,000 learners and demonstrated high participant satisfaction, strong knowledge acquisition, and widespread intent to implement IPC practices, reflecting positive outcomes across the first three levels of the Kirkpatrick Model. By providing no-cost, evidence-informed training aligned with CIC® core competencies, the program reduces barriers to professional development and supports statewide preparedness initiatives. Continued investment in virtual IPC education—paired with leadership engagement and expanded content offerings—may help sustain a resilient IPC workforce capable of responding to emerging infectious disease threats. The TEPHI IPC model offers a potential framework for other states and public health systems seeking to strengthen infection prevention capacity at scale.

## Data Availability

The raw data supporting the conclusions of this article will be made available by the authors, without undue reservation.
